# A novel zinc metabolism-related gene signature to predict prognosis and immunotherapy response in lung adenocarcinoma

**DOI:** 10.3389/fimmu.2023.1147528

**Published:** 2023-03-24

**Authors:** Wuguang Chang, Hongmu Li, Wei Ou, Si-Yu Wang

**Affiliations:** ^1^ State Key Laboratory of Oncology in South China, Collaborative Innovation Center for Cancer Medicine, Guangzhou, China; ^2^ Department of Thoracic Surgery, Sun Yat-sen University Cancer Center, Guangzhou, Guangdong, China

**Keywords:** zinc, zinc metabolism, biomarker, prognosis, lung adenocarcinoma, immunotherapy

## Abstract

**Background:**

Zinc is a key mineral element in regulating cell growth, development, and immune system. We constructed the zinc metabolism-related gene signature to predict prognosis and immunotherapy response for lung adenocarcinoma (LUAD).

**Methods:**

Zinc metabolism-associated gene sets were obtained from Molecular Signature Database. Then, the zinc metabolism-related gene signature (ZMRGS) was constructed and validated. After combining with clinical characteristics, the nomogram for practical application was constructed. The differences in biological pathways, immune molecules, and tumor microenvironment (TME) between the different groups were analyzed. Tumor Immune Dysfunction and Exclusion algorithm (TIDE) and two immunotherapy datasets were used to evaluate the immunotherapy response.

**Results:**

The signature was constructed according to six key zinc metabolism-related genes, which can well predict the prognosis of LUAD patients. The nomogram also showed excellent prediction performance. Functional analysis showed that the low-risk group was in the status of immune activation. More importantly, the lower risk score of LUAD patients showed a higher response rate to immunotherapy.

**Conclusion:**

The state of zinc metabolism is closely connected to prognosis, tumor microenvironment, and response to immunotherapy. The zinc metabolism-related signature can well evaluate the prognosis and immunotherapy response for LUAD patients.

## 1 Introduction

Lung cancer is the thorniest cancer in the world, and LUAD is the most prevalent type of non-small cell lung cancer (NSCLC), accounting for about 40% of lung cancer (1). In the past decades, with the increasing understanding of the molecular level, the treatment of lung cancer has undergone profound changes. Surgery or radiation is still the primary treatment for the early stage of lung cancer, while patients with advanced lung cancer have a variety of treatment options, including targeted treatment, immunotherapy, and combined with other therapies (2). Among them, immunotherapy brings great benefits to patients with advanced lung cancer. However, due to the heterogeneity of tumors in different individuals, about 70% of patients with advanced non-small cell lung cancer did not respond to immunotherapy (3). Therefore, the search for new targets still needs to be continued.

Zinc is one of the essential trace elements in the body (4). It is a key component of a variety of enzymes and transcription factors, including oxidoreductase, hydrolase, lyase, synthetase, and ligase, which are involved in regulating DNA synthesis and cell cycle (5, 6). Therefore, the lack of zinc will lead to the disorder of DNA replication process, and then cause the cell to lose control of proliferation and become cancerous. The zinc content in the serum of various cancer patients has been significantly reduced, including breast cancer (7), lung cancer (8), head and neck cancer (9), and prostate cancer (10). In addition, zinc widely affects the immune system of the body (11). Yu et al. found that the influx of zinc increased after T cell receptor (TCR) was initially stimulated, and then accelerated the activation of T cell by lowering the threshold of TCR activation, thereby enhancing the immune response (12). Various zinc finger proteins participate in the regulation of B cell maturation (13, 14). The deficiency of zinc transporter SLC39A10 will cause the decrease of zinc level in macrophages, which leads to p53 mediated apoptosis (15). Zinc deficiency also promotes the production of inflammatory cytokines IL-1β, IL-6, and TNF-α *via* the MAPK pathway (16). In view of the extensive role of metals such as iron and copper in cancer and the cell death they cause (ferroptosis and cuproptosis) (17), we believe that regulating the content of zinc in the body and targeting the key enzymes in zinc metabolism are expected to become new targets for cancer treatment in the future.

In this study, we constructed ZMRGS, which can accurately predict the prognosis of LUAD patients. It can also well evaluate the immunological characteristics and immunotherapy response of different LUAD patients.

## 2 Materials and methods

### 2.1 Data collection and processing

Transcriptome data of LUAD from TCGA was used for the training set. To reduce the impact of non-tumor factors, we excluded the lack of survival data and the samples with OS less than 30 days. Finally, we collected 485 LUAD and 59 normal samples. In addition, we also collected the copy number variation (CNV) data of LUAD in the TCGA database for analysis. The GSE72094 containing 398 LUAD from GEO was used as the validation set. Zinc metabolism-related genes were gathered from the molecular signature database (18).

### 2.2 Identification of differently expressed zinc metabolism-related genes and enrichment analysis

The differentially expressed zinc metabolism-related genes between tumor and normal tissues in the TCGA dataset were analyzed by ‘limma’ package (19). The screening criteria were set as adjusted p value < 0.05 and log2 |Fold Change| ≥ 1, which were displayed by heatmap and volcano plot. Subsequently, GO/KEGG enrichment analysis was performed on these genes through the ‘clusterProfiler’ package (20). The ‘CNBplot’ package is a tool for Bayesian network inference of enrichment analysis results (21). We used it to infer the gene interaction of key pathways.

### 2.3 Construction and validation of the zinc metabolism-related gene signature

To build a reliable prognostic signature, we first screened prognostic genes through univariate cox regression analysis and then incorporated them into the LASSO cox regression model to further reduce the number of candidate genes. Finally, the ZMRGS was constructed by multivariate cox regression analysis. The risk score of each LUAD sample = Coef_gene1_*Exp_gene1_+Coef_gene2_*Exp_gene2_ … Coef_genen_*Exp_genen_. According to the median risk score, LUAD patients were divided into high- and low-risk groups. KM survival analysis was used to analyze the survival differences between the two groups. Then the risk score of each LUAD patient in GSE72094 was calculated using the same algorithm and the same analysis was performed.

### 2.4 Nomogram for clinical application

Through cox regression analysis, we explored whether clinical factors affected risk score, and the results were shown by forest plot. To establish a clinically feasible scoring system, based on the results of cox regression analysis, we combined the risk score and the clinical information (p < 0.05) and constructed the nomogram. The reliability of the nomogram was verified by receiver operating characteristic (ROC) curve and calibration curve.

### 2.5 Gene set enrichment analysis

With the purpose of finding out the reasons that affect the survival differences between two risk groups, we used GSEA software (version: 4.2.3) to study the biological functions and pathways of the two subgroups. The threshold is set to p < 0.05 and false discovery rate (FDR) < 0.25.

### 2.6 Analysis of tumor environment features

Further to explore the differences in the characteristics of immune molecules in different subgroups, we extensively analyzed a variety of immune molecules, including chemokine, immunostimulator, immunoinhibitor, MHC molecule, and receptor. The correlation between key immune checkpoints and risk score was analyzed by spearman correlation analysis. Then, we used ssGSEA to analyze the composition of 28 kinds of immune cells in the TME (22). The ‘estimate’ package was used to analyze the differences of tumor microenvironment components as a whole (23).

### 2.7 Prediction of immunotherapy response

The TIDE algorithm is a method to calculate the dysfunction and exclusion of patients to immunotherapy according to the gene expression profile (24). The higher TIDE score means that patients are more prone to immune escape when receiving immunotherapy. We calculated the TIDE score of each LUAD patient and analyzed the response of different subgroups to immunotherapy through chi-square test. In order to further verify the predictive efficacy of zinc metabolism-related gene signature, we used two immunotherapy datasets with complete transcriptome data to verify (1) IMvigor210: a cohort of 298 patients with advanced urothelial carcinoma undergoing anti-PD-L1 (atezolizumab) immunotherapy (25). (2) Checkmate: a cohort of 181 patients with advanced renal cell carcinoma undergoing anti-PD-1 (nivolumab) immunotherapy (26).

### 2.8 Analysis of drug sensitivity

Based on the Cancer Genome Project (CGP) 2016 data in the ‘pRRophetic’ package (27), we analyzed the sensitivity of several commonly used chemotherapy drugs and compared the differences between the two groups.

### 2.9 Quantitative real-time PCR (qRT-PCR)

The expression profiles of 6 key genes were verified by quantitative real-time PCR (qRT-PCR). There were 16 paired LUAD and lung tissues used for experiment, which were from patients undergoing lung cancer resection at our center. This study was approved by the Ethics Committee of Sun Yat-sen University Cancer Center (YB2018-85). A TRIzol (TIANGEN, Beijing, China) was used to isolate total RNA from cancer tissue samples and adjacent normal tissue samples. PrimeScriptTM RT Master Mix (ES Science, Shanghai, China) was used to reverse-transcribe complementary DNA. SYBR Green Master Mix (ES Science, Shanghai, China) was used to amplify the target gene. Repeat the qRT-PCR assays for three times in 10ml reaction volume for each sample. The PCR primers used for amplification were as follows: *ABCC8*, 5′- TCACCTCCGTGGTCTACTATC -3′ (forward),5′- CTTGGTCTGTATTGCTCCTCTC -3′ (reverse); *CPS1*, 5′- CAACCTGGCAGTTCCTCTATAC-3′ (forward), 5′- ACAGCGTCCATTTCTACTTCTC -3′ (reverse); *HMGA2*, 5′- CAGGAAGCAGCAG-CAAGAA -3′ (forward),5′- CCAGGCAAGGCAACATTGA -3′ (reverse); *HVCN1*, 5′- TGCCTGGAACATCAACTACAA -3′ (forward), 5′- CTCCAGGCGGAAGACAAATAA -3′ (reverse); *MT1A*, 5′- CGCCTTATAGCCTCTCAACTTC -3′ (forward),5′- TAAATGGGTCAGGGTTGTATGG -3′ (reverse); *SLC39A11*, 5′- CAGCTCTCGTGTTCGTATTCTC -3′ (forward), 5′- AGGATGCCAGTTTCCCATTAC -3′ (reverse); GAPDH, 5′-GATTCCACCCATGGCAAATTC-3′ (forward), 5′- GTCATGAG-TCCTTCCACGATAC -3′ (reverse). By the comparative threshold cycle (2-Ct) method, we calculated the relative expression of these 6 genes by paired test.

### 2.10 Statistical analysis

All graphs and data analysis were conducted by R software (version: 4.13) and SPSS (version: 26). Chi-square test was used for categorical variables. The continuous variable was used by the Wilcoxon test.

## 3 Results

### 3.1 Differently expressed Zinc metabolism-related genes and function enrichment

**Figure 1:**
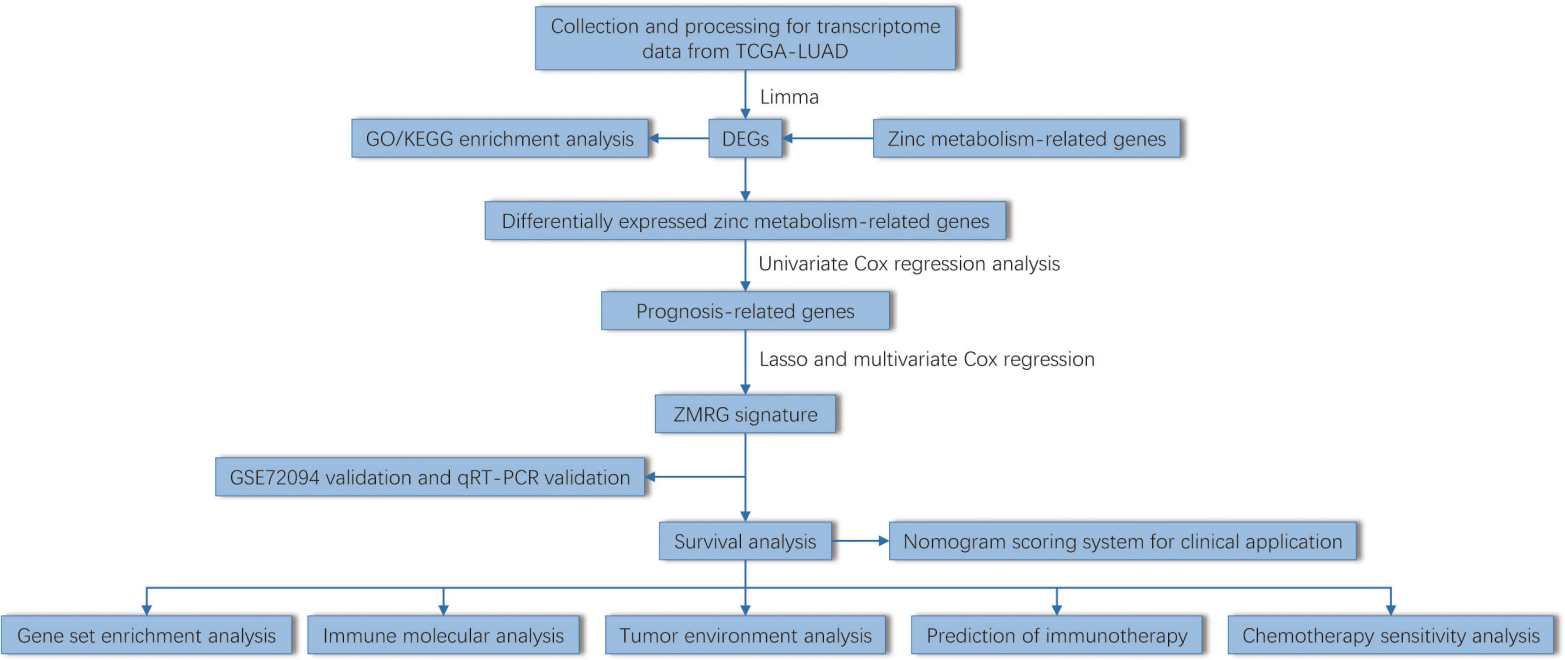
Flowchart in this study.

**Figure 2:**
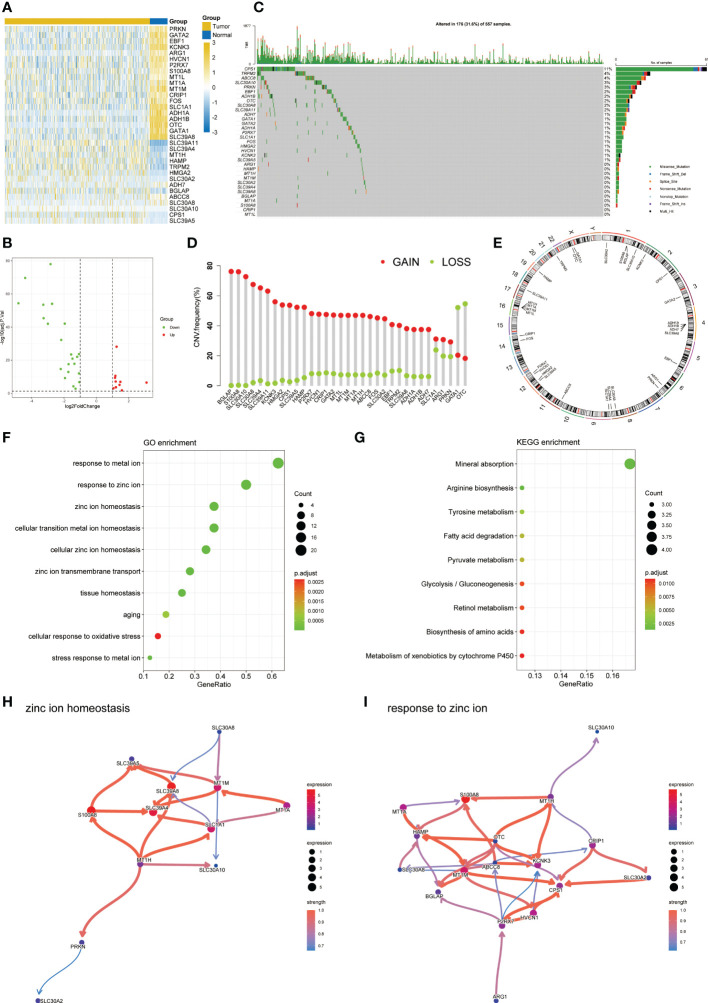
Characteristic of zinc metabolism-related genes in LUAD. **(A)** Heatmap showing the differences of zinc metabolism-related genes in LUAD and normal samples. **(B)** Volcano plot exhibiting 22 down-regulated and 11 up-regulated genes. **(C)** Gene mutation landscape of LUAD in TCGA. **(D)** The CNV mutation frequency of 33 zinc metabolism-related genes. **(E)** Chromosome position and alteration of zinc metabolism-related genes. **(F)** GO enrichment analysis. **(G)** KEGG enrichment analysis. Gene interaction network diagram in zinc ion homeostasis **(H)** and response to zinc ion **(I)**.

The flow chart of this study was shown in **Figure 1**. By analyzing the transcriptome differences between LUAD and normal tissues, we obtained 33 differentially expressed zinc metabolism-related genes (**Figure 2A**), of which 11 were up-regulated and 22 were down-regulated (**Figure 2B**). Based on the TCGA database, 31.6% (176/557) of the samples had gene mutations (**Figure 2C**), of which *CPS1* mutation rate was the highest, reaching 11%. In addition, the proportion of CNV gain of most genes was higher, only the results of *GATA1* and *OTC* were opposite (**Figure 2D**). The position of 33 genes on the chromosome was shown in **Figure 2E**. GO/KEGG enrichment analysis results showed that these genes were mainly concentrated in zinc metabolism activities, zinc homeostasis, and metabolism of various nutrients (**Figures 2F, G** **)**. Then, we conducted the Bayesian network analysis on the key zinc metabolism pathway (zinc homeostasis and response to zinc) and showed the interaction between genes (**Figures 2H, I** **)**.

### 3.2 Construction and validation of the zinc metabolism-related gene signature

**Figure 3:**
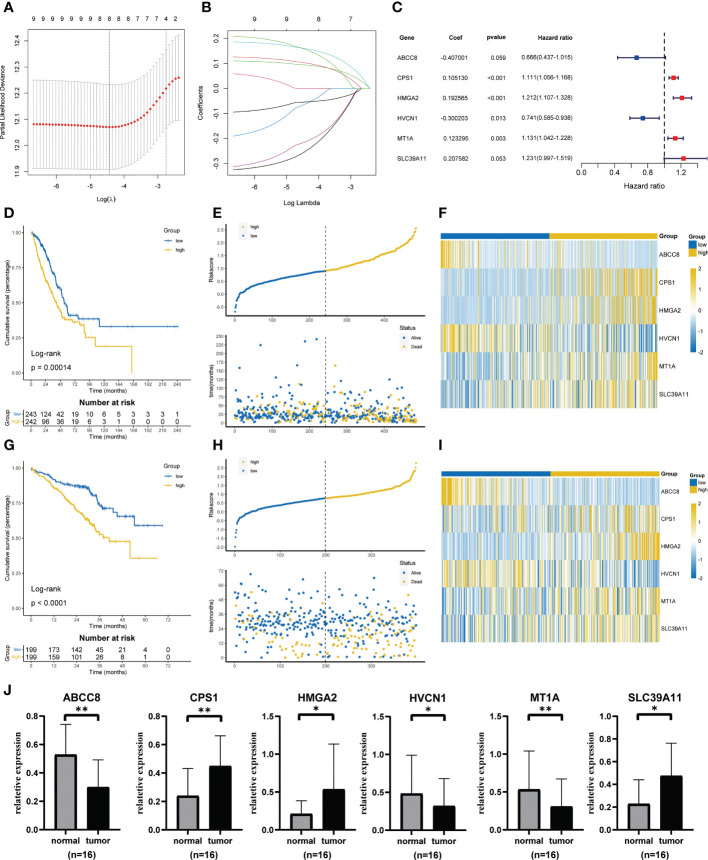
Construction and validation of ZMRGS. **(A)** Tenfold cross-validation in LASSO model. **(B)** LASSO coefficients of 9 prognostic-related genes. **(C)** 6 key zinc metabolism-related genes and their coefficients. **(D)** Kaplan-Meier survival analysis in TCGA cohort. **(E)** Distribution of risk score and OS status in TCGA cohort. **(F)** Heatmap of 6 zinc metabolism-related genes in TCGA cohort. **(G)** Kaplan-Meier survival analysis in GSE72094**. (H)** Distribution of risk score and OS status in GSE72094. **(I)** Heatmap of 6 zinc metabolism-related genes in GSE72094. **(J)** The mRNA expressions of ABCC8, CPS1, HMGA2, HVCN1, MT1A and SLC39A11 by qRT-PCR. *p < 0.05, **p < 0.01.

Univariate cox regression found that 9 genes were associated with prognosis (**Figure S1**). Based on the minimal lambda value of LASSO regression, we identified 8 genes (**Figures 3A, B** **)**. Then, six key genes and their coefficients were obtained through multivariate cox analysis (**Figure 3C**). Risk score = -ABCC8*0.407001+CPS1*0.105130+HMGA2*0.192565-HVCN1*0.300203+MT1A*0.123295+SLC39A11*0.207582. KM survival analysis showed that the prognosis of low-risk group was better (p = 0.00014, **Figure 3D**). The distribution of risk scores showed that more people in high-risk groups died (**Figure 3E**). **Figure 3F** showed the expression of six key genes. In addition, similar results were obtained in GSE72094 (**Figures 3G–I**). The qRT-PCR showed that a high expression of *CPS1*, *HMGA2*, and *SLC39A11* was found in tumor compared with adjacent normal tissues by 16 paired LUAD samples from our center, while the expression of *ABCC8*, *HVCN1*, and *MT1A* was lower in tumor (**Figure 3J**).

### 3.3 Establishment of the nomogram

**Figure 4:**
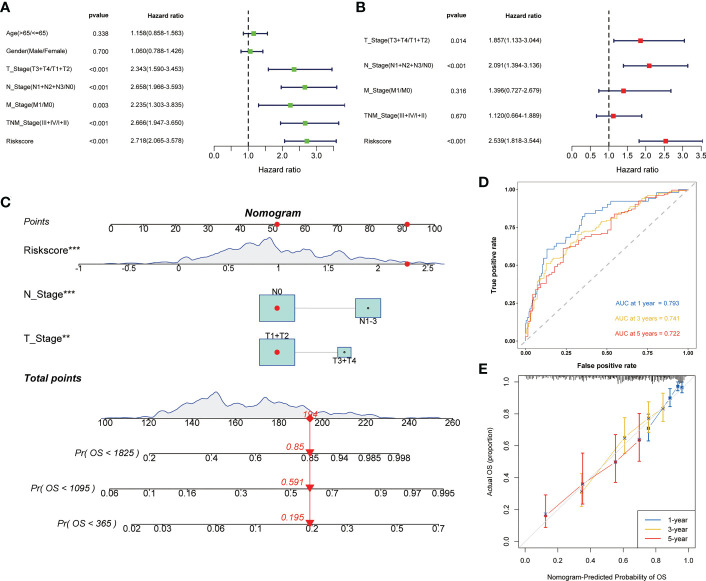
Construction of the nomogram to predict the prognosis of LUAD. Univariate **(A)** and multivariate **(B)** Cox regression analysis of risk score. **(C)** Nomogram for the prediction of 1-, 3- and 5-year survival probability. **(D)** Time-dependent ROC analysis of the nomogram. **(E)** Calibration curves for evaluating the accuracy. **p < 0.01, ***p < 0.001.

Univariate and multivariate cox regression analysis all showed that risk score was an independent risk factor affecting the prognosis of LUAD (**Figures 4A, B** **)**. Then, based on the above results, we built the nomogram to provide the basis for clinical practice (**Figure 4C**). The area under curve (AUC) values in 1, 3, and 5 years were 0.793, 0.741, and 0.722 respectively (**Figure 4D**). The correction curve also showed that the predicted value was consistent with the actual results (**Figure 4E**).

### 3.4 Potential functions and pathways in different subgroups

**Figure 5:**
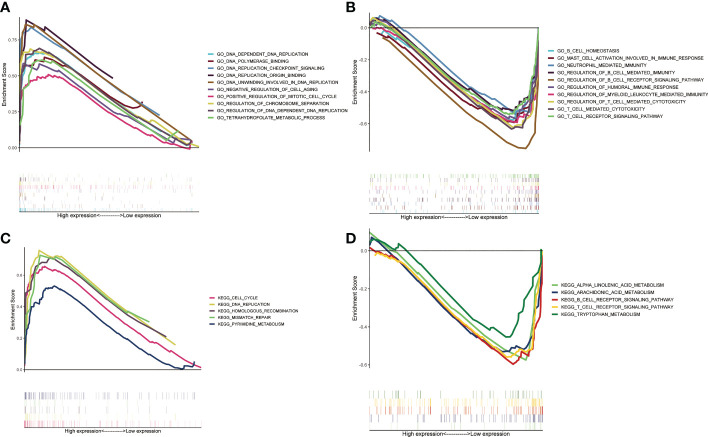
Gene set enrichment analysis between two ZMRGS groups. GO enrichment in high-risk group **(A)** and low-risk group **(B)**. KEGG enrichment in high-risk group **(C)** and low-risk group **(D)**.

Based on GSEA, we analyzed the functional and pathway differences between different subgroups. GO enrichment analysis showed that there were a large number of pathways related to cell proliferation and cell cycle regulation in high-risk group (**Figure 5A**). In the low-risk group, a large number of immune-related pathways were activated (**Figure 5B**). The results of KEGG enrichment analysis also confirmed the above conclusions (**Figures 5C, D** **)**.

### 3.5 Immune molecule analysis

**Figure 6:**
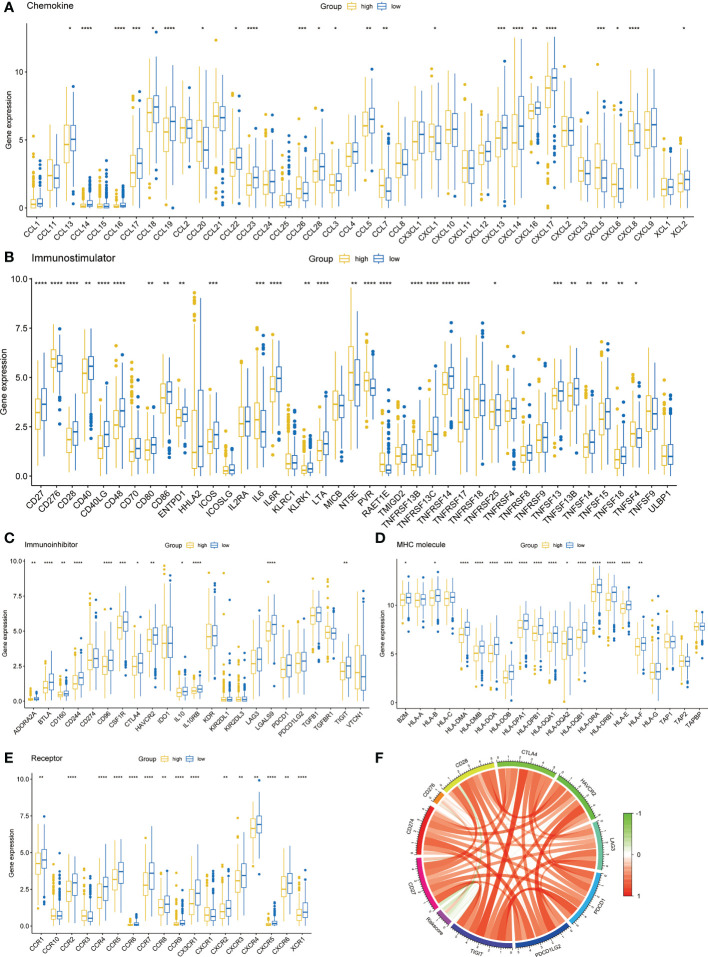
Immune-related molecular characteristics. **(A)** Chemokine. **(B)** Immunostimulator. **(C)** Immunoinhibitor. **(D)** MHC molecule. **(E)** Receptor. **(F)** Correlation circle of risk score and immune checkpoints. *p < 0.05, **p < 0.01, ***p < 0.001, ****p < 0.0001.

To investigate the differences of immune molecules between different subgroups, we systematically analyzed the differences between chemokine, immunostimulator, immunoinhibitor, MHC molecule, and receptor (**Figures 6A–E**). There were obvious differences in the expression of most immune molecules, and almost all immune molecules were more abundant in the low-risk group. Subsequently, we also analyzed the correlation between several key immune checkpoints and the risk score. The results showed that except CD276, the other immune checkpoints were negatively correlated with risk score, while the immune checkpoints were positively correlated (**Figure 6F**).

### 3.6 Differences of the tumor environment

**Figure 7:**
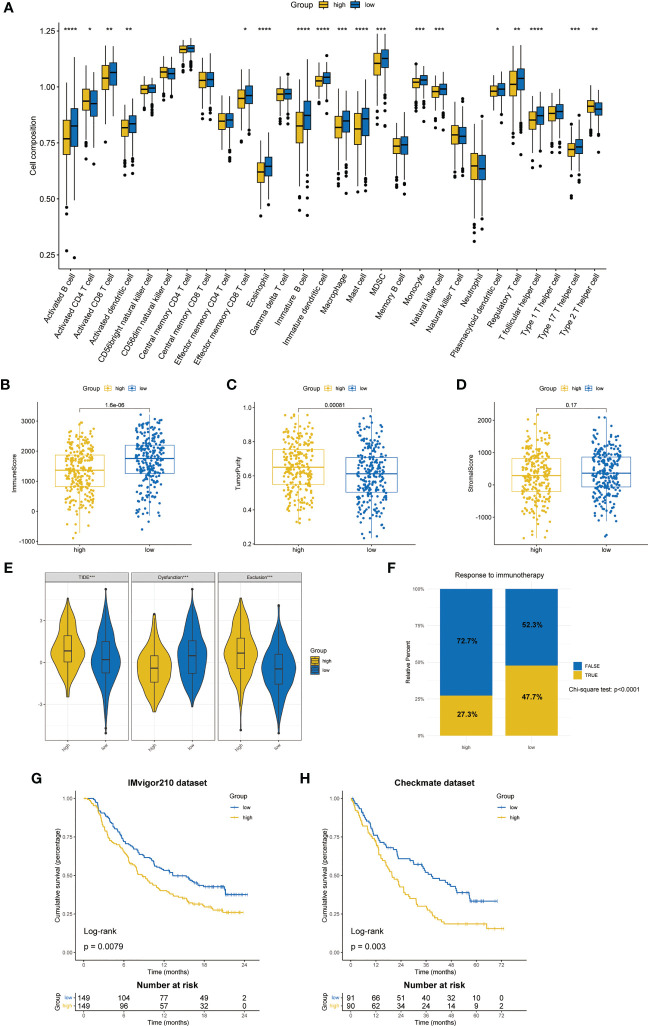
Differences in TME and prediction of immunotherapy response. **(A)** The differences in the proportions of 28 immune cells between two ZMRGS groups by ssGSEA. Results of immune score **(B)**, tumor purity **(C)**, and stromal score **(D)** in two groups. **(E)** The results of TIDE score. **(F)** Response to immunotherapy from TIDE algorithm. Kaplan–Meier analysis by ZMRGS for patients in the IMvigor210 cohort **(G)** and Checkmate cohort **(H)**. *p < 0.05, **p < 0.01, ***p < 0.001, ****p < 0.0001.

Based on ssGSEA algorithm, we found that up to 16 kinds of immune cells were significantly infiltrated in the low-risk group (57.1%), while only CD4 T cell and type 2 T helper cell were significantly infiltrated in the high-risk group (7.14%) (**Figure 7A**). The ESTIMATE algorithm showed that the low-risk group had a higher immune score (**Figure 7B**) and a lower tumor purity (**Figure 7C**). However, there was no significant difference between the stromal score (**Figure 7D**).

### 3.7 Prediction of immunotherapy response by TIDE and immunotherapy datasets

The results of TIDE suggested that the high-risk group had the higher TIDE score and exclusion score, while the low-risk group had higher dysfunctional score (**Figure 7E**), which meant that the high-risk group patients were more prone to immune escape. The result of response to immunotherapy also showed that the low-risk group was more likely to be responsive to immunotherapy (chi-square test, p < 0.0001, **Figure 7F**). In addition, we conducted KM survival analysis by using two independent immunotherapy datasets, and the results showed that the prognosis of high-risk patients was worse (IMvigor210: p = 0.0079, Checkmate: p = 0.003, **Figures 7G, H** **)**.

### 3.8 Analysis of drug sensitivity

**Figure 8:**
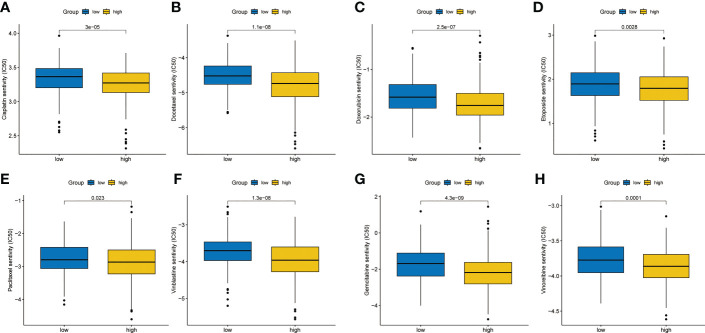
Chemotherapy sensitivity analysis. **(A)** Cisplatin. **(B)** Docetaxel. **(C)** Doxorubicin. **(D)** Etoposide. **(E)** Paclitaxel. **(F)** Vinblastine. **(G)** Gemcitabine. **(H)** Vinorelbine.

According to the data from Cancer Genome Project (CCP) 2016 in the ‘pRRophetic’ package, we analyzed the relative sensitivity of various drugs. The overall drug sensitivity analysis results were shown in **Figure S2**. Surprisingly, the high-risk group was more sensitive to common drugs (**Figures 8A–H** **)**.

## 4 Discussion

Research on the connection between zinc and cancer has made some hopeful strides in recent years. Zinc widely affects the proliferation of tumor cells and the function of immune system (28), more and more studies have proved that zinc plays an irreplaceable role in the occurrence and development of tumors. However, the study of key genes in zinc metabolism is rare. As far as we know, this is the first study to analyze the prognostic value and immune characteristics of zinc metabolism-related genes in LUAD.

In this study, we analyzed the genomic changes of zinc metabolism-related genes in LUAD and constructed the ZMRGS. The signature showed superior prediction ability. To get closer to clinical practice, we combined the signature with TNM staging to construct the nomogram. The prognostic signature of this study was composed of six genes (*ABCC8*, *CPS1*, *HMGA2*, *HVCN1*, *MT1A*, and *SLC39A11*). Previous studies had described the partial relationship between these genes and tumors. *ABCC8* is a member of the MRP family involved in multidrug resistance. It was regarded as a prognostic marker for glioma and could predict chemosensitivity (29). *CPS1* was thought to be involved in metabolic reprogramming in hepatocellular carcinoma, thus affecting the occurrence of tumors (30). The development of inhibitors for *CPS1* also shows potential therapeutic potential (31). The high expression of *HMGA2* in lung cancer indicates a worse prognosis, which is consistent with our findings. Vivo experiments have confirmed that overexpression of *HMGA2* can inhibit the proliferation and metastasis of lung cancer, so *HMGA2* is expected to become a new target for the treatment of lung cancer (32). *HVCN1* is the only gene encoding mammalian proton channel. Zinc ion can promote cell apoptosis and inhibit the invasion and metastasis of glioma cells by inhibiting the activity of *HVCN1 in vivo* and *in vitro*. *MT1A* is involved in DNA damage reaction and metal homeostasis and is therefore inseparable from the occurrence and development of tumors (33). *SLC39A11* is a zinc transporter, which can inhibit the cloning of LUAD cells after being knocked down *in vitro (* 34).

Zinc is involved in the formation of various enzymes that regulate cell cycle. Lack of zinc will lead to abnormal cell proliferation and then carcinogenesis. Through GSEA analysis, we found that a variety of cell cycle-related pathways were activated in high-risk group, which may be the reflection of zinc regulation of cell cycle disorder leading to poor prognosis of LUAD patients in our model. In addition, both non-specific and specific immune systems are affected by zinc, involving the proliferation, differentiation, and apoptosis of immune cells (35). Our research also found that there were a large number of immune pathways enriched and higher expression of immune molecules in the low-risk group, indicating that the low-risk group immune pathway was activated. The results of ssGSEA also confirmed that various immune cells infiltrated in the low-risk group. These results indicate that the low-risk group was in the state of immune activation on the whole. Abundant immune cell infiltration means that it is more likely to display a better response to immunotherapy (36). Subsequently, we predicted the response to immunotherapy through the TIDE algorithm. The results showed that, as we expected, the high-risk group had a higher TIDE score and was more prone to immune escape and the low-risk group showed excellent immunotherapy response (47.7% vs 27.3%). To verify the reliability of the results of TIDE, we used two datasets of anti-PD-1/PD-L1 immunotherapy for further analysis. The results, without exception, suggest that patients in the low-risk group had better prognosis when receiving immunotherapy. Although great progress has been made in immunotherapy, only 20% of patients with NSCLC respond to immune checkpoint inhibitors (ICIs) (37). Previous studies have confirmed that ICIs combined with chemotherapy could improve the effectiveness of immunotherapy (38); hence, we also analyzed the sensitivity of chemotherapy drugs and found that the high-risk group was more sensitive to multiple chemotherapy drugs. Therefore, immunotherapy combined with chemotherapy for the high-risk group may be an option to improve the prognosis of such patients. These results provided a reliable basis for our ZMRGS to be applied in clinical practice and provide different treatment guidance for different LUAD populations.

There are some limitations in this study. First of all, the data we analyzed was from the public database, and further multi-center clinical trial verification is still needed in the future. Secondly, due to the lack of transcriptome datasets of LUAD immunotherapy, the prediction of immunotherapy was only based on some machine algorithms. Finally, the potential role of these key zinc metabolism-related genes in lung cancer still needs further basic experimental exploration.

In conclusion, we have constructed a new and reliable zinc metabolism-related gene signature, which can effectively predict the prognosis of LUAD patients and distinguish the immune status of different LUAD patients. More importantly, it can distinguish and give LUAD patients with different immune states more appropriate treatment to improve their prognosis.

## Data availability statement

The datasets presented in this study can be found in online repositories. The names of the repository/repositories and accession number(s) can be found in the article/**Supplementary Material**.

## Ethics statement

This study was approved by the Ethics Committee of Sun Yat-sen University Cancer Center (YB2018-85). The patients/participants provided their written informed consent to participate in this study. Written informed consent was obtained from the individual(s) for the publication of any potentially identifiable images or data included in this article.

## Author contributions

WC and HL: Conceptualization, data curation, formal analysis, writing–original draft, writing–review and editing. WO: data-collecting, formal analysis, writing–review and editing. S-YW: Conceptualization, supervision, funding acquisition, writing–original draft, project administration, writing–review, and editing. All authors contributed to the article and approved the submitted version.

## Supplementary material

Supplementary Figure 1 Univariate cox regression analysis of 33 zinc metabolism-related genes.

Supplementary Figure 2 Sensitivity of TCGA-LUAD patients to chemotherapy drugs.

## References

[1] Siegel RL Miller KD Fuchs HE Jemal A. Cancer statistics, 2021. CA Cancer J Clin (2021) 71(1):7–33. doi: 10.3322/caac.21654 33433946

[2] Miller M Hanna N. Advances in systemic therapy for non-small cell lung cancer. BMJ (2021) 375:n2363. doi: 10.1136/bmj.n2363 34753715

[3] Mamdani H Matosevic S Khalid AB Durm G Jalal SI. Immunotherapy in lung cancer: Current landscape and future directions. Front Immunol (2022) 13:823618. doi: 10.3389/fimmu.2022.823618 35222404 PMC8864096

[4] Saper RB Rash R. Zinc: an essential micronutrient. Am Fam Physician (2009) 79(9):768–72. PMC2820120 20141096

[5] Ho E. Zinc deficiency, DNA damage and cancer risk. J Nutr Biochem (2004) 15(10):572–8. doi: 10.1016/j.jnutbio.2004.07.005 15542347

[6] Dreosti IE. Zinc and the gene. Mutat Res (2001) 475(1-2):161–7. doi: 10.1016/S0027-5107(01)00067-7 11295161

[7] Jouybari L Kiani F Akbari A Sanagoo A Sayehmiri F Aaseth J. A meta-analysis of zinc levels in breast cancer. J Trace Elem Med Biol (2019) 56:90–9. doi: 10.1016/j.jtemb.2019.06.017 31442959

[8] Scheiermann E Puppa MA Rink L Wessels I. Zinc status impacts the epidermal growth factor receptor and downstream protein expression in A549 cells. Int J Mol Sci (2022) 23(4). doi: 10.3390/ijms23042270 PMC8876057 35216384

[9] Buntzel J Bruns F Glatzel M Garayev A Mucke R Kisters K. Zinc concentrations in serum during head and neck cancer progression. Anticancer Res (2007) 27(4A):1941–3. 17649800

[10] Goel T Sankhwar SN. Comparative study of zinc levels in benign and malignant lesions of the prostate. Scand J Urol Nephrol (2006) 40(2):108–12. doi: 10.1080/00365590500368922 16608807

[11] Prasad AS. Zinc in human health: effect of zinc on immune cells. Mol Med (2008) 14(5-6):353–7. doi: 10.2119/2008-00033.Prasad PMC2277319 18385818

[12] Yu M Lee WW Tomar D Pryshchep S Czesnikiewicz-Guzik M Lamar DL. et al: Regulation of T cell receptor signaling by activation-induced zinc influx. J Exp Med (2011) 208(4):775–85. doi: 10.1084/jem.20100031 PMC3135340 21422171

[13] Dinkel A Warnatz K Ledermann B Rolink A Zipfel PF Burki K. The transcription factor early growth response 1 (Egr-1) advances differentiation of pre-b and immature b cells. J Exp Med (1998) 188(12):2215–24. doi: 10.1084/jem.188.12.2215 PMC2212439 9858508

[14] Jurado S Gleeson K O'Donnell K Izon DJ Walkley CR Strasser A. The zinc-finger protein ASCIZ regulates b cell development *via* DYNLL1 and bim. J Exp Med (2012) 209(9):1629–39. doi: 10.1084/jem.20120785 PMC3428950 22891272

[15] Gao H Zhao L Wang H Xie E Wang X Wu Q. et al: Metal transporter Slc39a10 regulates susceptibility to inflammatory stimuli by controlling macrophage survival. Proc Natl Acad Sci USA (2017) 114(49):12940–5. doi: 10.1073/pnas.1708018114 PMC5724256 29180421

[16] Wessels I Haase H Engelhardt G Rink L Uciechowski P. Zinc deficiency induces production of the proinflammatory cytokines IL-1beta and TNFalpha in promyeloid cells *via* epigenetic and redox-dependent mechanisms. J Nutr Biochem (2013) 24(1):289–97. doi: 10.1016/j.jnutbio.2012.06.007 22902331

[17] Tong X Tang R Xiao M Xu J Wang W Zhang B. Targeting cell death pathways for cancer therapy: Recent developments in necroptosis, pyroptosis, ferroptosis, and cuproptosis research. J Hematol Oncol (2022) 15(1):174. doi: 10.1186/s13045-022-01392-3 36482419 PMC9733270

[18] Liberzon A Birger C Thorvaldsdottir H Ghandi M Mesirov JP Tamayo P. The molecular signatures database (MSigDB) hallmark gene set collection. Cell Syst (2015) 1(6):417–25. doi: 10.1016/j.cels.2015.12.004 PMC4707969 26771021

[19] Ritchie ME Phipson B Wu D Hu Y Law CW Shi W. Limma powers differential expression analyses for RNA-sequencing and microarray studies. Nucleic Acids Res (2015) 43(7):e47. doi: 10.1093/nar/gkv007 25605792 PMC4402510

[20] Yu G Wang LG Han Y He QY. clusterProfiler: an r package for comparing biological themes among gene clusters. OMICS (2012) 16(5):284–7. doi: 10.1089/omi.2011.0118 PMC3339379 22455463

[21] Sato N Tamada Y Yu G Okuno Y. CBNplot: Bayesian network plots for enrichment analysis. Bioinformatics (2022) 38(10):2959–60. doi: 10.1093/bioinformatics/btac175 PMC9113354 35561164

[22] Hanzelmann S Castelo R Guinney J. GSVA: gene set variation analysis for microarray and RNA-seq data. BMC Bioinf (2013) 14:7. doi: 10.1186/1471-2105-14-7 PMC3618321 23323831

[23] Yoshihara K Shahmoradgoli M Martinez E Vegesna R Kim H Torres-Garcia W. et al: Inferring tumour purity and stromal and immune cell admixture from expression data. Nat Commun (2013) 4:2612. doi: 10.1038/ncomms3612 24113773 PMC3826632

[24] Jiang P Gu S Pan D Fu J Sahu A Hu X. Signatures of T cell dysfunction and exclusion predict cancer immunotherapy response. Nat Med (2018) 24(10):1550–8. doi: 10.1038/s41591-018-0136-1 PMC6487502 30127393

[25] Mariathasan S Turley SJ Nickles D Castiglioni A Yuen K Wang Y. TGFbeta attenuates tumour response to PD-L1 blockade by contributing to exclusion of T cells. Nature (2018) 554(7693):544–8. doi: 10.1038/nature25501 PMC6028240 29443960

[26] Braun DA Hou Y Bakouny Z Ficial M Sant' Angelo M Forman J. Interplay of somatic alterations and immune infiltration modulates response to PD-1 blockade in advanced clear cell renal cell carcinoma. Nat Med (2020) 26(6):909–18. doi: 10.1038/s41591-020-0839-y PMC7499153 32472114

[27] Geeleher P Cox N Huang RS. pRRophetic: an r package for prediction of clinical chemotherapeutic response from tumor gene expression levels. PLoS One (2014) 9(9):e107468. doi: 10.1371/journal.pone.0107468 25229481 PMC4167990

[28] Skrajnowska D Bobrowska-Korczak B. Role of zinc in immune system and anti-cancer defense mechanisms. Nutrients (2019) 11(10). doi: 10.3390/nu11102273 PMC6835436 31546724

[29] Zhou K Liu Y Zhao Z Wang Y Huang L Chai R. ABCC8 mRNA expression is an independent prognostic factor for glioma and can predict chemosensitivity. Sci Rep (2020) 10(1):12682. doi: 10.1038/s41598-020-69676-7 32728190 PMC7391768

[30] Cancer Genome Atlas Research Network. Electronic address wbe, cancer genome atlas research n: Comprehensive and integrative genomic characterization of hepatocellular carcinoma. Cell (2017) 169(7):1327–41.e1323. doi: 10.1016/j.cell.2017.05.046 28622513 PMC5680778

[31] Zhang L Zou Y Lu Y Li Z Gao F. Unraveling the therapeutic potential of carbamoyl phosphate synthetase 1 (CPS1) in human diseases. Bioorg Chem (2023) 130:106253. doi: 10.1016/j.bioorg.2022.106253 36356370

[32] Gao X Dai M Li Q Wang Z Lu Y Song Z. HMGA2 regulates lung cancer proliferation and metastasis. Thorac Cancer (2017) 8(5):501–10. doi: 10.1111/1759-7714.12476 PMC5582513 28752530

[33] Si M Lang J. The roles of metallothioneins in carcinogenesis. J Hematol Oncol (2018) 11(1):107. doi: 10.1186/s13045-018-0645-x 30139373 PMC6108115

[34] Wang S Gui P Liu Y Liang X Fan B Shang W. Role of methylation-related genes CRYAB and SLC39A11 in the occurrence and development of lung adenocarcinoma. Ann Transl Med (2022) 10(20):1126. doi: 10.21037/atm-22-3576 36388803 PMC9652513

[35] Bonaventura P Benedetti G Albarede F Miossec P. Zinc and its role in immunity and inflammation. Autoimmun Rev (2015) 14(4):277–85. doi: 10.1016/j.autrev.2014.11.008 25462582

[36] Emens LA Silverstein SC Khleif S Marincola FM Galon J. Toward integrative cancer immunotherapy: Targeting the tumor microenvironment. J Transl Med (2012) 10:70. doi: 10.1186/1479-5876-10-70 22490302 PMC3341195

[37] Doroshow DB Herbst RS. Treatment of advanced non-small cell lung cancer in 2018. JAMA Oncol (2018) 4(4):569–70. doi: 10.1001/jamaoncol.2017.5190 29494728

[38] Gandhi L Rodriguez-Abreu D Gadgeel S Esteban E Felip E De Angelis F. et al: Pembrolizumab plus chemotherapy in metastatic non-Small-Cell lung cancer. N Engl J Med (2018) 378(22):2078–92. doi: 10.1056/NEJMoa1801005 29658856

