# Supplementary figures and images for: A novel zinc metabolism-related gene signature to predict prognosis and immunotherapy response in lung adenocarcinoma
Source: Front Immunol. 2023 Mar 24;14:1147528. doi: 10.3389/fimmu.2023.1147528 (PMC10079938; doi:10.3389/fimmu.2023.1147528)

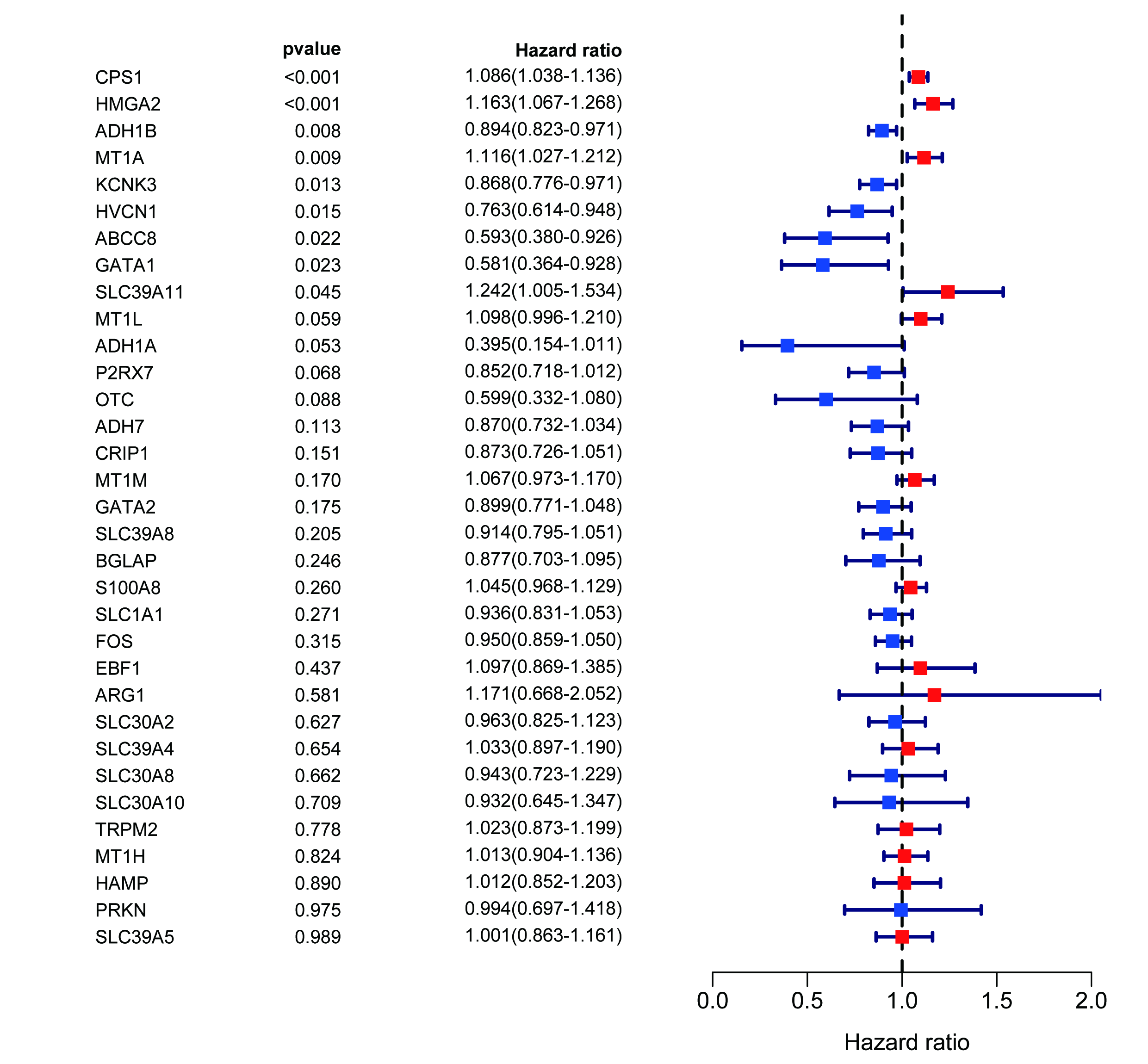

Supplement: Supplementary Figure 1 — Univariate cox regression analysis of 33 zinc metabolism-related genes. [file Image_1.tif]

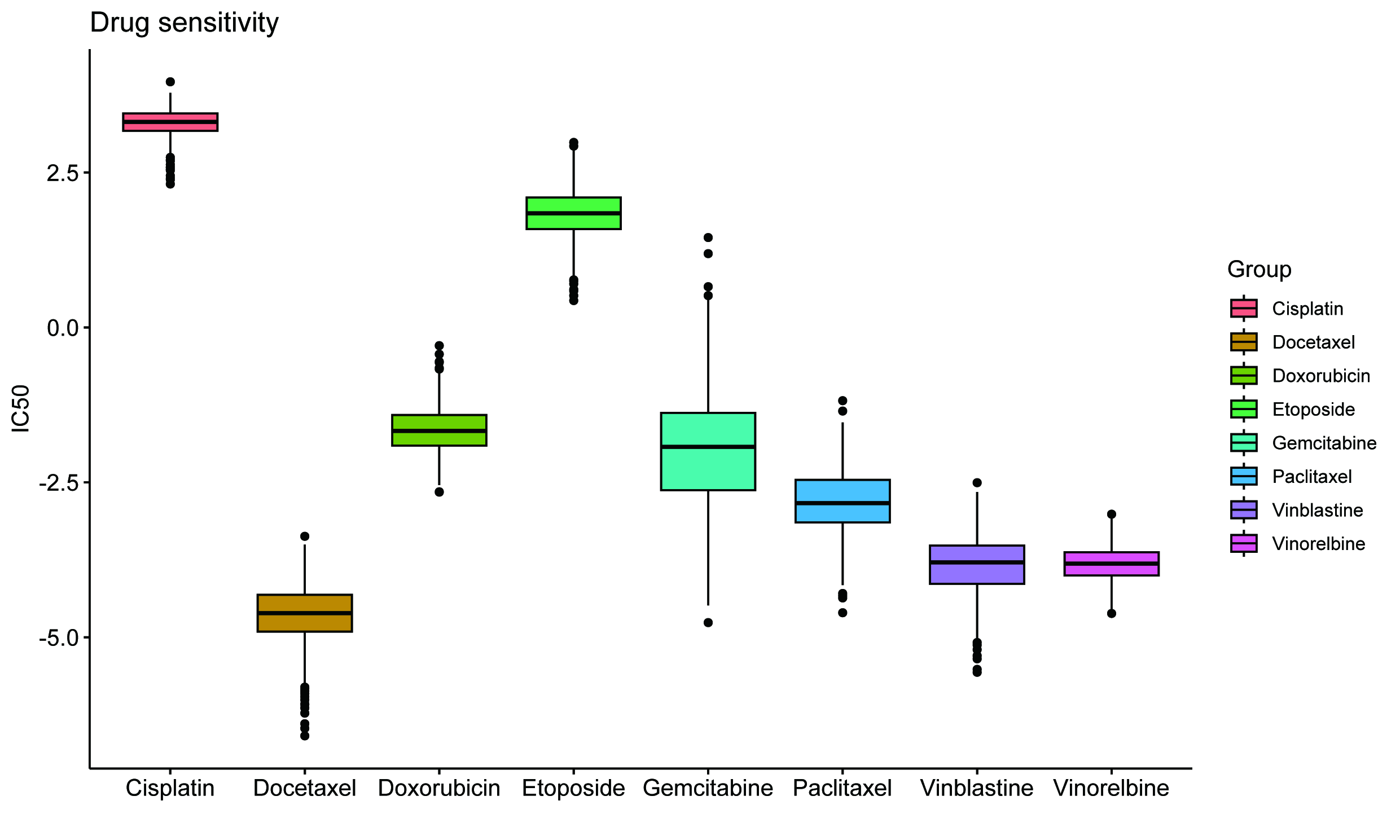

Supplement: Supplementary Figure 2 — Sensitivity of TCGA-LUAD patients to chemotherapy drugs. [file Image_2.tif]
